# Experimental dataset of enhanced rheological properties and lubricity of Nigerian bentonite mud using kelzan® xcd polymer and identifying it optimal combination

**DOI:** 10.1016/j.dib.2018.06.047

**Published:** 2018-06-22

**Authors:** Tomiwa Oguntade, Oluwatosin Rotimi, Aroyehun Mojisole, Adenubi Solomon, Gambo Angye

**Affiliations:** Department of Petroleum Engineering, Covenant University, Ota, Nigeria

## Abstract

The experimental dataset in this article are for improved rheological properties and lubricity of Nigerian bentonite mud using Kelzan® xcd polymer and identifying it optimal combination. For this study, water base mud was formulated using a Nigerian bentonite and a statistical based method was used to analyze the rheological and lubricity properties of the drilling fluid, when enhanced with kelzan® xcd polymer. The significant and interaction level of these factors were closely observed on the mud properties test that were conducted. The use of response surface design was engaged to analyze the influence of bentonite quantity and the quantity of kelzan® xcd polymer on the lubricity and rheological properties of the mud. Minitab 17 (Minitab Inc. USA) was used for the response surface design. The *p*-values were used to determine which of the factors in the model are statistically significant, which was compared to α-level (0.05). The *p*-values for the quantity of kelzan® xcd polymer are 0, 0, and 0.007 for Apparent viscosity (cp), Yield point (Ib/100 ft^2), Plastic viscosity (cp) respectively. All these values are lesser than the α-level (0.05), which means that the effect of kelzan® xcd polymer is significant on the model. While the effect of Bentonite content and the interaction between Bentonite content and kelzan® xcd polymer are insignificant because their *p*-values are higher than the -level (0.05).

**Specifications Table**

**Table:** 

Subject area	*Petroleum Engineering*
More specific subject area	*Drilling fluids*
Type of data	*Tables and figures*
How data was acquired	*Data were collected from laboratory tests using OFITE Viscometer Model 800 and OFITE lubricity tester*
Data format	*Raw and analyzed*
Experimental factors	*1. Local bentonite used for this study was sourced from Abeokuta Ogun state in Nigeria*
	*2. Water base mud was formulated, using different grams of bentonite and kelzan® xcd polymer*
	*3. Rheological and lubricity properties test were conducted using OFITE Viscometer Model 800 and OFITE lubricity tester*
Experimental features	*Improving the rheological and lubricity properties of water based drilling fluid, when a local bentonite and kelzan® xcd polymer are used for the formulation.*
Data source location	*Department of Petroleum Engineering, Covenant University, Nigeria*
Data accessibility	*All data are available in the article*

**Value of the Data**

• The addition of high amount of kelzan® xcd polymer will improve the rheological properties and lubricity of Nigerian bentonite.
• The data shows that there were significant changes in the rheological properties of water based mud, when different concentration of kelzan® xcd was added.
• A statistical tool like Minitab 17 (Minitab Inc. USA), can be used in the industry to manage quality analysis and process improvement in other to make more effective business decisions.
• The optimal combination of bentonite and kelzan® xcd polymer was obtained from the data using response optimizer.

## 1 Data

**Fig. 1:**
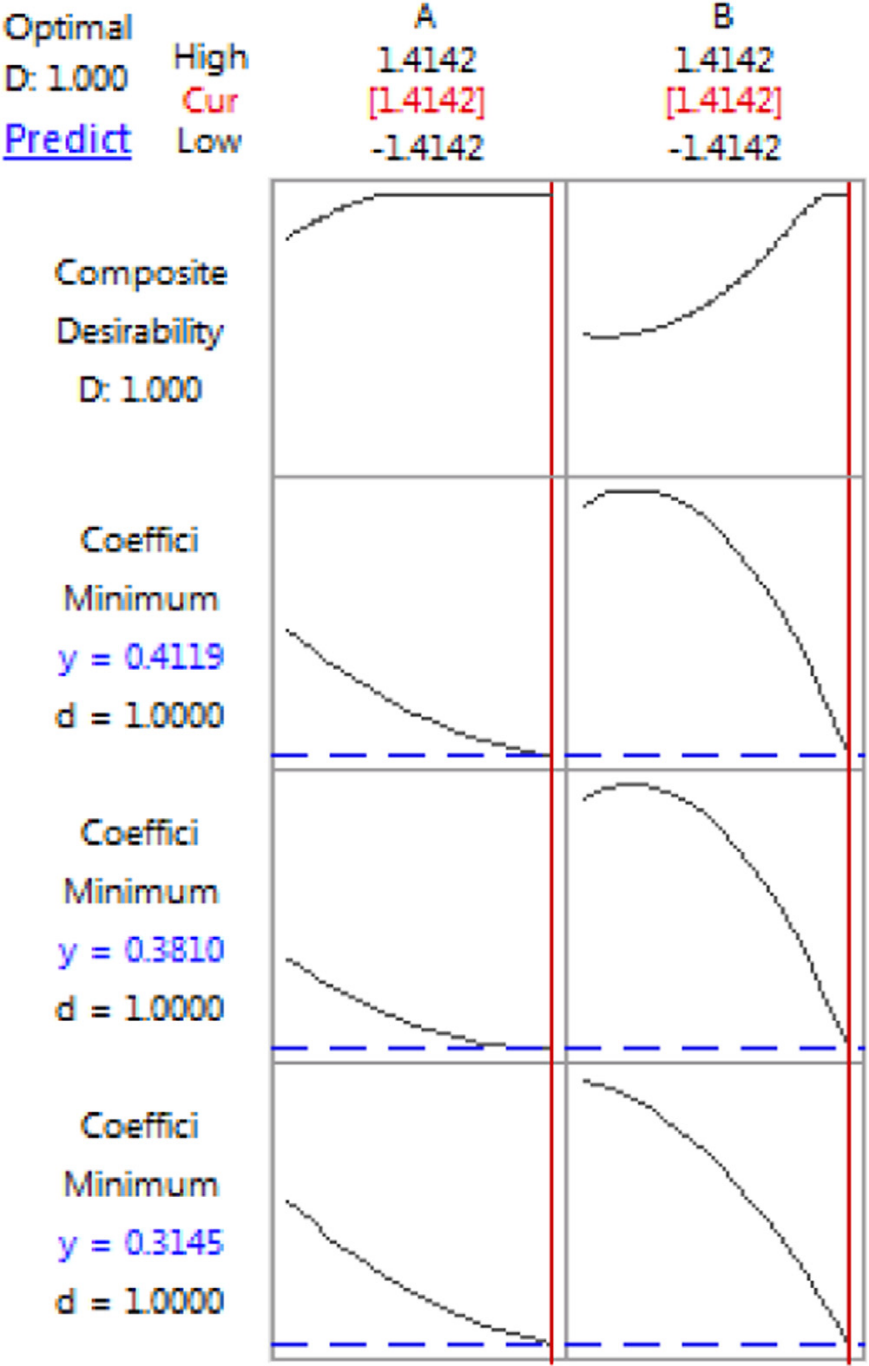
Response optimization plot.

The petroleum industry is a major prospective consumer of Nigerian Bentonite, although Nigerian bentonite have various important uses in several part of industrial fields. Therefore there is a need to enhance the properties of the clay to meet up with required standards, for formulation of drilling mud in petroleum industry Response surface design was used to analyze the influence of bentonite quantity and the quantity of kelzan® xcd polymer on the rheological properties and lubricity of the drilling fluid. Minitab 17 was use for the response surface design. Table 1 shows the highest and lowest factor ranges for bentonite and kelzan® xcd polymer concentration, used in the formulation of the mud. Table 2 shows the response design and the rheological properties of the enhanced drilling mud. Table 3 shows the effects, *p*-value and regression coefficients for the rheological properties (yield point, plastic viscosity and apparent viscosity) gotten from response surface analysis in Minitab 17. Table 4 gives the response design and the Lubricity result of the enhanced drilling mud. Table 5 shows the effects, regression coefficients and *p*-value for coefficient of friction @60 RPM, coefficient of friction @200 RPM and coefficient of friction @600 RPM. Table 6 gives the response Optimization Parameters. Table 7 shows the response Optimization Solution. Whereas Fig. 1 displays the response optimization plot.

**Table 1:** Factor range.

Factors	Representation	Unit	Level	
			Low	High
Bentonite quantity	A	grams	20	28
kelzan® xcd polymer	B	grams	0.2	1

**Table 2:** The response design and the rheological properties.

RunOrder	A^a^	B^a^	Bentonite content^b^	kelzan® xcd polymer^b^	Plastic viscosity(cp)	Yield point(Ib/100 ft^2),	Apparent viscosity(cp)
1	−1.41421	0	20	0.6	6	18	15
2	0	1.414214	24	1	15	53	41.5
3	0	0	24	0.6	11	22	22
4	−1	1	22	0.8	15	49	39.5
5	0	0	24	0.6	11	22	22
6	0	0	24	0.6	11	23	22.5
7	1	−1	26	0.4	11	17	19.5
8	1	1	26	0.8	18	45	40.5
9	0	0	24	0.6	11	22	22
10	−1	−1	22	0.4	9	20	19
11	0	−1.41421	24	0.2	9	13	15.5
12	1.414214	0	28	0.6	11	34	28
13	0	0	24	0.6	8	28	22

a Variables in coded levels

b Un-coded variables.

**Table 3:** Effects, *p*-value and regression coefficients for the rheological properties (yield point, plastic viscosity and apparent viscosity).

	Apparent viscosity(cp)			Yield point(Ib/100 ft^2)			Plastic viscosity(cp)		
Term	Estimated Effect	Regression Coefficient	*P*-Value	Estimated Effect	Regression Coefficient	*P*-Value	Estimated Effect	Regression Coefficient	*P*-Value
Constant	–	22.1	0		23.4	0		10.4	0
A	4.97	2.49	0.078	3.91	1.95	0.283	3.018	1.509	0.071
B	19.57	9.78	0	28.39	14.2	0	5.371	2.686	0.007
A*A	1.71	0.86	0.529	4.22	2.11	0.279	−0.4	−0.2	0.801
B*B	8.71	4.36	0.012	11.23	5.61	0.017	3.1	1.55	0.081
A*B	0.25	0.13	0.944	−0.5	−0.25	0.919	0.5	0.25	0.811

**Table 4:** The response design and the Lubricity result.

RunOrder	A^a^	B^a^	Bentonite content^b^	kelzan® xcd polymer^b^	Coefficient of Friction @60 RPM	Coefficient of Friction @200 RPM	Coefficient of Friction @600 RPM
1	1	−1	26	0.4	0.398	0.468	0.488
2	0	0	24	0.6	0.389	0.479	0.519
3	0	0	24	0.6	0.392	0.462	0.492
4	1	1	26	0.8	0.3409	0.4109	0.4409
5	−1.41421	0	20	0.6	0.448	0.528	0.558
6	0	−1.41421	24	0.2	0.432	0.502	0.532
7	−1	1	22	0.8	0.352	0.412	0.452
8	0	1.414214	24	1	0.3391	0.3991	0.4291
9	0	0	24	0.6	0.3852	0.4652	0.4852
10	0	0	24	0.6	0.391	0.461	0.481
11	1.414214	0	28	0.6	0.375	0.455	0.475
12	0	0	24	0.6	0.392	0.482	0.512
13	−1	−1	22	0.4	0.4	0.48	0.52

a Variables in coded levels.

b Un-coded variables.

**Table 5:** Effects, regression coefficients and *p*-value for Coefficient of Friction @60 RPM, Coefficient of Friction @200 RPM and Coefficient of Friction @600 RPM.

	Coefficient of Friction @60 RPM			Coefficient of Friction @200 RPM			Coefficient of Friction @600 RPM		
Term	Estimated Effect	Regression Coefficient	*P*-Value	Estimated Effect	Regression Coefficient	*P*-Value	Estimated Effect	Regression Coefficient	*P*-Value
Constant	–	0.38984	0		0.46984	0		0.49784	0
A	−0.02908	−0.01454	0.065	−0.02908	−0.01454	0.09	−0.04012	−0.02006	0.029
B	−0.05912	−0.02956	0.003	−0.06766	−0.03383	0.003	−0.06516	−0.03258	0.003
A*A	0.00876	0.00438	0.558	0.00751	0.00375	0.65	0.00701	0.0035	0.67
B*B	−0.01719	−0.00859	0.266	−0.03344	−0.01672	0.073	−0.02894	−0.01447	0.109
A*B	−0.00455	−0.00227	0.815	0.0054	0.0027	0.802	0.0105	0.0052	0.63

**Table 6:** Response optimization parameters.

**Response**	**Goal**	**Lower**	**Target**	**Upper**	**Importance**
Coefficient of Friction @600 RPM	Minimum	–	0.4291	0.558	1
Coefficient of Friction @200 RPM	Minimum	–	0.3991	0.528	1
Coefficient of Friction @60 RPM	Minimum	–	0.3391	0.448	1

**Table 7:** Response optimization solution.

			Coefficient of Friction @600 RPM	Coefficient of Friction @200 RPM	Coefficient of Friction @60 RPM	Composite
Solution	A*	*B	Fit	Fit	Fit	Desirability
1	1.41421	1.41421	0.411919	0.380954	0.31449	1

## 2 Experimental design, materials and methods

### 2.1 Nigerian bentonite

The local bentonite used for this study was sourced from Abeokuta Ogun state in Nigeria. The bentonite which are made up of mostly montmorillonite, which is a division of the smectites group. (Mg[Si_4_O_10_] × [OH]_2_) × p([Al, Fe]_2_ × [Si_4_O_10_]) is the common expression for montmorillonite mineral that exist in a bentonite.

### 2.2 kelzan® xcd polymer

Kelzan® xcd polymer was acquired from Equilab Business Solutions Limited in Lagos state Nigeria. The kelzan® xcd polymer was manufactured by Diversity Technologies Corp and have the following physical and chemical properties; a white – tan powder appearance, it׳s soluble in water and has a pH of 7.0 (in a 1% solution).

### 2.3 Design of experiment

A 2^2^ (2-Level, 2-Factors) central composite design was used to create a statistical model to study the quadratic effects and interaction effects between the kelzan® xcd polymer and bentonite particles. MINITAB® 17 (PA, USA) statistical software was used in the design of experiment and statistical analysis of the experimental data [1].

### 2.4 Rheological prosperities measurements

An OFITE Viscometer Model 800 which has eight regulated test speeds of 3 Gel, 6 RPM, 30 RPM, 100 RPM, 200 RPM, 300 RPM and 600 RPM was used in determining the flow characteristics of the enhanced Nigerian bentonite when kelzan® xcd polymer was applied. A control knob is use to easily change the speed of the viscometer and the lighted magnified dial displays the dial reading. The API standard procedure 13B-1 and 13B-2 were used for the calibration of the viscometer, following the equipment standard calibration.

### 2.5 Lubricity measurement

The drilling fluid lubricating quality and lubricating additive fluid resistance was measured by an OFITE lubricity tester. A 150 in – pounds of force which is the standard lubricity coefficient test was applied between the two hardened steel surfaces, a ring rotating at 60, 200, 600 RPM and a block. Friction was measured as the coefficient of friction (*μ*). The coefficient of friction between two solids is defined as the frictional force of the load or the force perpendicular to the surfaces.
