# Supplementary material for: Experimental dataset of enhanced rheological properties and lubricity of Nigerian bentonite mud using kelzan® xcd polymer and identifying it optimal combination
Source: Data Brief. 2018 Jun 22;19:1804–9. doi: 10.1016/j.dib.2018.06.047 (PMC6141262; doi:10.1016/j.dib.2018.06.047)
Supplement: Supplementary file 1 — Supplementary material [file mmc1.docx]

I confirm that this work is original and has not been published elsewhere nor is it currently under consideration for publication elsewhere.
